# Increased antimicrobial use during COVID‐19: The risk of advancing the threat of antimicrobial resistance

**DOI:** 10.1002/hsr2.459

**Published:** 2021-12-14

**Authors:** Mahesh Jampani, Sujith J. Chandy

**Affiliations:** ^1^ International Water Management Institute (IWMI ‐ CGIAR) Battaramulla Colombo Sri Lanka; ^2^ Department of Pharmacology and Clinical Pharmacology Christian Medical College Vellore India

The repeated waves of the novel coronavirus disease (COVID‐19) across the world have resulted in major issues in different facets of life, especially in healthcare. In low‐ and middle‐income countries (LMICs) such as India, the effects of the pandemic have been devastating, driving communities, hospitals, and government into a crisis mode.^1^ There were a series of lockdowns and other pandemic‐related restrictions since March 2020, which appeared to have curbed the infection rates in the first wave. However, since April 2021, the number of people infected and deaths associated with the second wave of COVID‐19, specifically due to the delta variant, has been alarming. As a consequence, governments and scientific communities have been keen on finding an effective treatment for COVID‐19.

Many drugs were proposed to minimize morbidity and mortality, even though evidence regarding effectiveness was inconclusive. However, the fear among the public often heightened through the media, and the need for some form of treatment encouraged the rampant use of vitamins, immunity boosters, combination drugs or cocktail of antivirals, antibiotics, steroids, and antifungals.^2^, ^3^, ^4^ The popular antimicrobials frequently used include hydroxychloroquine, ivermectin, favipiravir, doxycycline, azithromycin, etc.^5^ Purchasing these antimicrobials require a prescription; however, community intake of these drugs surged with over‐the‐counter purchases, probably due to the desperate situation and the need to take some form of treatment. As a result, several antimicrobial drug sales skyrocketed, and many of them became out of stock in May 2021.^6^

Increased antimicrobial sales and consequent consumption during COVID‐19 might be due to various reasons and factors. These include: symptoms of COVID‐19, especially cough and fever, could be mistaken for a bacterial infection and vice versa; some studies have tried out several antimicrobials for COVID‐19 such as hydroxychloroquine, ivermectin, and azithromycin, and doctors, therefore, tend to prescribe^7^; many research studies though lacking conclusive evidence later become pieces of information which floated around in the news and social media, leading to purchase of these medicines by the public; as there is still no cure as per evidence, people are desperate and therefore take whatever they can, including antimicrobials. Such increased sales and intake of antimicrobials may directly contribute to increased antimicrobial resistance (AMR).

AMR has been recognized as a global public health emergency and has been on the agenda of the United Nations General Assembly (UNGA).^8^ Subsequent to the initiation of the global action plan on AMR and the work of the Tripartite Plus group (WHO, Food and Agriculture Organization of the United Nations [FAO], the World Organisation for Animal Health [OIE], and UNEP),^9^ there is an urgency in the light of increasing use of last‐resort antibiotics to save the lives of those who have bacterial infections. This problem is further compounded by the lack of research and development for newer classes of antibiotics. It is in this context, the increased sales of antimicrobials during the pandemic acquire a threatening dimension through the possible acceleration of the AMR phenomenon. It was therefore deemed important to determine whether there was an increased search interest for antimicrobials during the pandemic through the internet as a surrogate marker for increased sales and consumption among the public. We, therefore, did a brief analytical study of Google search trends for some of the antimicrobials that were reported to be commonly used by the public. Figure 1 shows the search interest of two popular antibiotics, azithromycin and doxycycline, in India.

**FIGURE 1:**
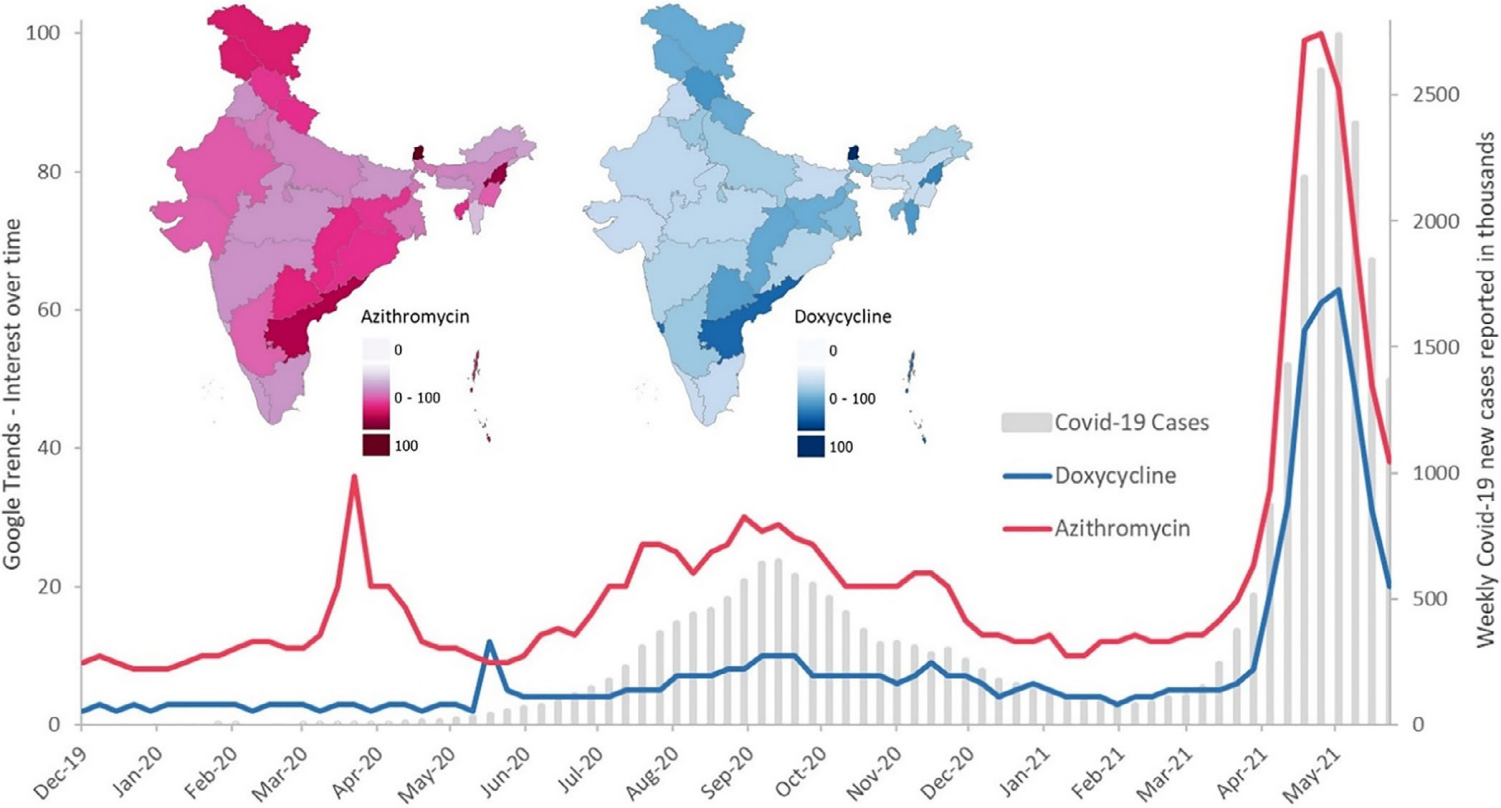
Google Trends results explaining web search interest over time in India (weekly data included from December 01, 2019 to May 29, 2021; https://trends.google.co.in) and subset maps explaining state‐wise search interest for respective antibiotics. Search interest explaining 100 signifies peak popularity for the antibiotics azithromycin and doxycycline.COVID‐19 cases data source: https://ourworldindata.org/coronavirus

The increased Google search trends for azithromycin and doxycycline in a close proximity with the beginning of the pandemic and the subsequent waves of the pandemic in July to November 2020 and April to May 2021 periods suggest a possible increase in antimicrobial drug consumption (Figure 1). This possible consumption may have become a behavioral practice to minimize the risks associated with COVID‐19 and save lives. This raises an important question: whether the treatment practices associated with the current pandemic crisis will further worsen the “silent pandemic” of AMR, its evolution in society and the environment.^10^

Even though the current COVID‐19 mortality rates appear very high, the faceless nature of AMR, its ever‐evolving nature, its multidimensional effects, and the complexity of arriving at solutions suggests that AMR would be a much bigger threat in terms of infection and death rates.^11^ It is well known that antibiotic use is a major contributing factor toward rising AMR.^12^ Consequently, antibiotic overuse and misuse during this pandemic and future ones need to be recognized and counter‐strategies devised to prevent a further rise in AMR.^4^ Recent research studies have shown that several antibiotics, especially doxycycline and azithromycin, are ineffective against treating COVID‐19.^13^ Though it is unknown how much the actual consumption increased during the pandemic, the Google Trends data showing the search interest are very high in India for doxycycline and azithromycin during the pandemic, especially between the months of April to May 2021. In particular, certain states have a high search interest for both antibiotics, thereby possibly suggesting an increased antibiotic consumption in these states (Figure 1).

To add another dimension, a recent study by Chakraborty and colleagues in Chennai in September 2020 detected high concentrations of azithromycin in wastewater, possibly reflecting its increased community consumption.^14^ Furthermore, during the COVID‐19 pandemic, overall antimicrobial consumption from the community and hospitals might be significantly higher.^4^, ^14^ Therefore, the total amount of antimicrobial loadings entering through hospital wastewater, human feces, and urine into the natural environment needs attention. The main concern is whether these potentially very large amounts of antimicrobial loadings within the last year will increase selection pressure in the natural environment and influence environmental bacteria to become resistant.

At the governance level, policymakers should be aware of these extremely large antimicrobial consumption patterns during the pandemic and take actions including awareness building for the public, guidelines for healthcare professionals, and overall stricter regulatory implementation to optimize antibiotic use and thereby mitigate future risk of infections associated with AMR. Over the years, antimicrobial resistance stewardship programs have helped to optimize prescribing antimicrobials, but these efforts need to be doubled during the pandemic and beyond. The brief analysis done with Google search trends suggests that coherent approaches are needed to understand and tackle the possibility of COVID‐19‐associated antimicrobial consumption and its impact on infectious disease patterns so as to prevent a future catastrophe through the faceless pandemic of AMR.

## FUNDING

Mahesh Jampani is supported by the Foreign, Commonwealth and Development Office (FCDO, UK) grant to the CGIAR research program on Agriculture for Nutrition and Health (A4NH) led by IFPRI and implemented through the CGIAR Antimicrobial Resistance Hub.

## CONFLICT OF INTEREST

On behalf of all authors, the corresponding author states that there is no conflict of interest.

## AUTHOR CONTRIBUTIONS

Conceptualization: Mahesh Jampani and Sujith J. Chandy.

Formal Analysis: Mahesh Jampani.

Visualization: Mahesh Jampani.

Writing—Original Draft: Mahesh Jampani.

Writing—Review and Editing: Mahesh Jampani and Sujith J. Chandy.

All authors have read and approved the final version of the manuscript.

The corresponding author had full access to all of the data in this study and takes complete responsibility for the integrity of the data and the accuracy of the data analysis.

## TRANSPARENCY STATEMENT

The corresponding author affirms that this manuscript is an honest, accurate, and transparent account of the study being reported; that no important aspects of the study have been omitted; and that any discrepancies from the study as planned have been explained.

## Data Availability

The authors confirm that the data supporting the findings of this study are available within the article.
